# Mortality in Extremely Low-Birth-Weight Neonates in México City (1985–2009) 

**DOI:** 10.1155/2010/265146

**Published:** 2010-12-21

**Authors:** José Iglesias-Leboreiro, Isabel Bernardez-Zapata, José Ramírez-Haua, Rocco González-Morán, Mario Enrique Rendón-Macías

**Affiliations:** ^1^Unidad de Cuidados Neonatales, Hospital Español de México, Ejercito Nacional 613, C-302 colonia Granada, CP 11520, Mexico; ^2^Departamento de Postgrado, Escuela de Medicina Universidad La Salle México, Fuentes #17, esquina Av. San Fernando, colonia Tlalpan, CP 14000, Mexico; ^3^Unidad de Investigación en Epidemiología Clínica, UMAE Hospital de Pediatría CMN siglo XXI, Instituto Mexicano del Seguro Social, Avenida Cuauhtemoc 330 colonia Doctores, CP 06720, Mexico

## Abstract

*Objective*. To analyze 25 years of mortality of extremely low-birth-weight (ELBW) neonates (≤1000 g) in a private hospital in Mexico City and to establish the current viability limit for ELBW neonates. *Methods*. We designed a prospective observational study of all ELBW neonates born between 1985 and 2009. Neonatal mortality, early neonatal mortality, and the 120-day mortality rate were analyzed in 5-year intervals by two categories of birth weight (501–750 g and 751–1000 g). *Results*. Among the 50,823 total births, 158 were ELBW (3.1 per 10^3^). Neonatal mortality (death ≤28 days) decreased for the 501–750 g neonates from 88.9% (1985–1989) to 55.6% (2005–1999) (*P* = .008) and for 751–1000 g neonates also decreased from 50% to 5.3% (*P* = .002). The 120-day mortality for neonates over 500 g diminished: 501–750 g neonates, 88.9% to 61.1% (*P* = .02) and for 751–1000 g neonates, 62.5% to 15.8% (*P* = .002). The highest viability limit was established in neonates who weighed ≥650 g and were ≥26 weeks in gestational age. *Conclusions*. The survival of ELBW neonates has improved in Mexico particularly in private hospitals, and it was more evident over the years 2004–2009. These data suggest that it is possible to increase the ELBW neonates survive in developing counties.

## 1. Introduction 

Advances in prenatal care and the availability of specialized centers have resulted in higher survival rates and a reduction in medium- and long-term complications for extremely low-birth-weight (ELBW) neonates [1, 2]. Favorable changes have been observed since the beginning of the 21st century due to the incorporation of prenatal strategies such as the use of prenatal corticoids, exogenous surfactants, different types of ventilation, better control of nosocomial infections, and early enteral feeding strategies [3]. In 2005, Itabashi et al. [1] reported an 87% 28-day survival of ELBW neonates and an 83% rate of discharge from the Neonatal Intensive Care Unit (NICU). 

Regardless of these achievements, the increase in survival of ELBW neonates may be attributed to the presence of medical center facilities with neonatal care. In a recent Japanese study, the authors reported a 4.9-fold increased risk of death for a neonate born and treated in a medical facility outside of Tokyo, especially in centers that delivered 10 or less neonates per year [1]. The Neonatal Research Network (NICHD) suggested that the 120-day survival ranged from 38% to 76% for neonates who weighed 501–750 g and ranged from 74% to 94% for those who weighed 750–1000 g [4]. 

The most influential factors for survival may be the equipment in the NICUs and the skills of the personnel who handle the newborns [5, 6]. 

Few studies have reported on the survival of ELBW neonates in developing countries. In India, 501–750 g neonates have a 23% NICU survival and 751–1000 g neonates have a 61% survival [7]; in Thailand, 501–750 g neonates have a 20% 28 days survival, and 751–1000 g neonates have a 62% 28 days survival. Interestingly, in both countries, the mortality rate for neonates weighing less than 500 g was 100% [8]. 

In 2000, Latin American countries saw neonatal mortality (<28 days) for 500–999 g neonates decrease from 95% to 68% [9]. The <28 days survival for 500–750 g neonates improved from 19.1% to 21.8%, and more importantly, the survival for 751–1000 g neonates improved from 73.4% to 75% [10, 11]. Interestingly, the survival differed by the type of facility; as Matijasevich et al. [12] described, the survival for 500–999 g neonates was 16.7% in public facilities and 53.2% in private facilities.

Mexico has few specialized centers for the care of ELBW neonates. Most of them are Social Security centers, which are either partially or completely funded by the government. Neonatal survival has been reported in only one of those centers: in 1999–2001, the <28 survival rate was 52.6% for ELBW neonates, including those weighing less than 500 g; in 2003 the survival rate was 46% for the 600–1000 g neonates [10]. Also, this information is limited in time and of poor quality, and information from private hospitals is lacking. 

The quality of health care varied for neonates in private medical centers in Mexico. Hospital Español de México is a tertiary care private facility with gynecology/obstetrics and perinatology services as well as specialized clinics for high-risk pregnancies, infertility, and assisted reproductive technology. The center also has a state-of-the-art NICU. This hospital receives the financial support of the Sociedad de Beneficencia Española (Spanish Beneficence Society). In addition, it is a teaching hospital that trains nursing personnel and medical residents in pediatrics, neonatology, and other specialties. The main objective of this study was to determine the mortality rate trends in 5-year periods from 1985 to 2009 for ELBW neonates at this NICU. In addition, to provide better information for parents and to perform objective evaluations to assist physicians in the decision-making process, we determined the viability limits in this facility. 

## 2. Methods

The study population comprised all neonates with a birth weight of 1000 g or less, regardless of the gestational age, born alive between January 1st, 1985, and December 31st, 2009, in the Hospital Español de México. At this institution, all newborns who fit the criteria were admitted to the NICU. Currently, the hospital's Ethics Committee supports neonatal resuscitation for all newborns with signs of life at birth. 

Once the neonates were stabilized, they were weighed using the same electronic scale (Tanita). The research team registered all events and comorbidities for the neonates during their stay at the hospital prior to discharge or death. This information was collected on a standardized form and then saved to a previously designed and codified electronic database. The stored data was periodically tested for reliability. The project was approved by the Hospital Research Committee.

## 3. Definitions

The mortality rate was calculated using the number of deaths that occurred among ELBW neonates during the first 28 days of life while at the hospital among the total number of ELBW newborns at the hospital. ELBW was defined as a birth weight 1,000 gram (g) or less. Neonatal early mortality rate was the ratio of the number of deaths during the first seven days of life to the total number of ELBW neonates. The 120-day mortality was calculated during the followup outside the hospital. All babies were reexamined monthly or revised during posterior hospitalization. Deaths were registered when parents notified to the research team or by telephone interview when someone missed a consultation, or when the baby died in hospital after a readmission. 

Gestational age at birth was obtained from the mother's last menstrual period (LMP) or by ultrasound.

## 4. Statistical Analysis

The mortality rates were analyzed for each 5-year period, and trends were assessed. The patients were categorized into three strata: less than 500 g, 501–750 g, and 751–1000 g. Mortality rates were calculated as percentages. 

To compare the rates among the 5-year periods, Chi-square tests linear-by-linear association were performed. The characteristics of the groups were compared using Pearson's Chi-square test. Box and Whisker plots were made for each 5-year period to compare the length of hospital stay among died, survival, or transferred patients. The nonparametric Kruskal-Wallis test was used to compare the length of hospitalization between the neonates who died and those who survived. A *P*-value <.05 was considered statistically significant. All analyses were performed with SPSS 13 (Chicago, Ill). 

To determine the variability limit in our hospital, we considered the combination of gestational age and the birth weight for which the 28-day survival after birth reached 50% or more of the neonates with these conditions.

## 5. Results

During the study period, 50,823 infants were born, and among them 158 were ELBW neonates, reflecting a prevalence of 3.1 per 10^3^ live newborns at the hospital (95% confidence interval [95% CI]: 2.6 to 3.6 per 10^3^). The prevalence was significantly higher (*P* < .001) during the most recent 5-year period; the rates varied from 3.3 per 10^3^ (95% CI: 2.2 to 4.3 per 10^3^) in 1985–1989 to 1.9 per 103 (95% CI: 1.07 to 2.7 per 10^3^) in 1990–1994, 1.8 per 10^3^ (95% CI: 0.8 to 2.7 per 10^3^) in 1995–1999, 2.5 per 10^3^ (95% CI: 1.3 to 3.7 per 10^3^) in 2000–2004, and 6.0 per 10^3^ (95% CI: 4.1 to 7.9 per 10^3^) in 2005–2009. 

Across the five-year periods, there were no differences in the newborns characteristics such as sex, type of pregnancy (single, twins, or multiple), or gestational age (Table 1).

## 6. Mortality Rate

Table 2 presents the early-neonatal, neonatal, and 120-day mortality rates by 5-year periods. The rates decreased across the periods, and this trend was statistically significant. The most notable decrease was the 38.3% reduction (from 62.8% to 24.5%) in the early-neonatal mortality rate; meanwhile, the neonatal and 120-day mortalities were reduced by approximately 34% between the first and last periods. 

This decrease in mortality was also observed among the different birth weight groups. None of the neonates weighing less than 500 g survived more than two days, representing 5% of the study population prior to 2005. In the last period, the 120 days survival of two neonates in that birth weight category was achieved. Mortality for the 501–750 g neonates decreased by 50% for early mortality and by 30% for the neonatal and 120-day mortality. The early mortality rate in the 751–1000 g neonates was reduced by 40%; the neonatal and 120-day mortalities decreased by 45% and 37%, respectively. Mortality differed among the 5-year periods (X2 = 17.18; 4 gl, *P* = .002). In the first two periods (1985–1989 and 1990–1994), mortality was over 80% in the early neonatal period (Table 2). Mortality was not statistically different between the 1995–1999 and 2005–2009 periods; mortality was predominantly higher in the early-neonate group (≤8 days) (U Mann Whitney, *P* = .58) although the last period had the lowest mortality rate (Table 2). Mortality in 2000–2004 was again associated with early-neonate group in eight patients: one was less than 500 g, five were in the 501–750 g range, and two were in the 751–1000 g range. In 2005–2009, among the 19 deceased patients, nine were early neonatal (56.2%), three were neonatal (18.7%), and four were postneonatal (25%).

## 7. Hospital Stay

**Figure 1:**
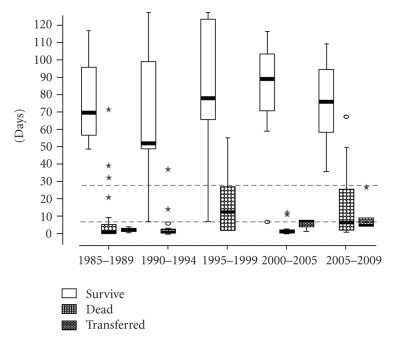
Length of hospital stay in the NICU by ELBW infants according to survival or death by 5-year periods.

Figure 1 shows that there were few changes in the length of hospital stay during the analyzed periods. For surviving patients, the mean length of stay in the hospital varied from 50 to 90 days. There were no statistically significant differences among the 5-year periods (Kruskal-Wallis test, X2 = 1.17; 4 gl, *P* = .88).

## 8. Transfers

Only six patients were transferred to other centers for neonatal care; there were no statistically significant differences regarding the length of hospital stay (Kruskal-Wallis, X2 = 5.45; 2 gl, *P* = .17).

## 9. Viability Limits

**Figure 2:**
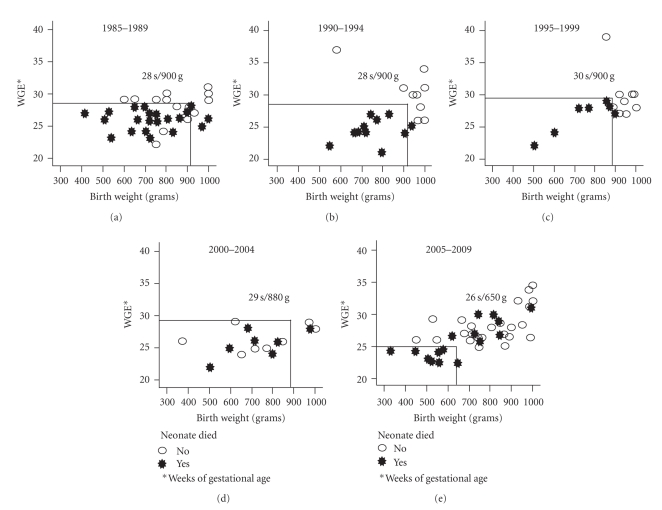
Viability margins by 5-year periods in relation to birth weight and gestational age.

Figure 2 presents the correlations between weight and gestational age of the neonates; these correlations are stratified by survival and by 5-year periods. Lines represent the survival limits, and it was observed that for the 1985–1989 period, the mortality was higher than 90% in neonates weighing less than 780 g who were born prior to week 28 of gestation. The limit of survival has been shortened through the different periods, especially after 2000. Currently, the viability limits may be established for neonates weighing less than 650 g that were born prior to week 25 of gestation. 

## 10. Discussion

To our knowledge, this is the first study that reports the mortality rates in ELBW neonates in a private hospital in Mexico. As mentioned above, the survival of these children is mainly attributed to the available resources, the expertise of the personnel, and the number of ELBW neonates cared for by the facility [1, 13, 14]. Our data describe the progressive achievements in our institution during the last few years and indicate the future needs and requirements to achieve better outcomes. Currently, we consider the survival of the babies born at our institution is similar to that reported in developed countries [2, 4, 13, 15, 16] (Table 3). 

Worldwide, ELBW survival may be evaluated with regard to birth weight or gestational age, [2] due to their impact on survival. However, birth weight, rather than gestational age has been recommended as the preferable predictor of survival because the gestational age of preterm infants is difficult to calculate. Therefore, as previous studies have suggested [2, 13], we designed our study to assess survival based mainly on the birth weight of infants. 

Similar to other reported rates worldwide, our hospital has seen an increase in ELBW and/or extremely premature neonates. Global reports suggest that prevalence of ELBW neonates increased from 0.3 to 2% of all newborn infants [4]. The increase in ELBW newborns has been evident since the 1980s and 1990s, with an estimated annual increase of 20%. At our institution, we have also seen a significant increase in ELBW neonates through the years, from (in the 1980s) 3.3 per 10^3^ live births to 6.0 per 10^3^ live births (in the last four years). This 45% increase is much higher than that published in studies from the US and Europe [4, 15, 16] and could be a consequence of several factors particular to our facilities which include the resuscitation of every newborn with or without signs of life, independently of the gestational age or birth weight. This practice has varied since 1985; in the mid-1990s, neonatal resuscitation programs were implemented. 

Another considerable factor is that our institution is a referral center for high-risk pregnancies, specializing in assisted pregnancies, under a joint agreement between the obstetrical and neonatology services. So, it is very important for us to ensure that every conception achieved has the opportunity to live, no matter the weight or gestational age. Lastly, the increase in the incidence of ELBW newborns may be partly related to the prevalence of multiple pregnancies, mainly as a consequence of assisted reproduction programs within the hospital.

Our data suggest a statistically significant clinical decrease in the ELBW infant mortality rate during the studied period, especially during the last 5-year period. Some of the major factors that may have contributed to this decrease were: the implementation of a training program for doctors and residents to learn how to resuscitate neonates, the use of exogenous surfactant and prenatal steroids, new modalities in assisted ventilation, the incorporation of nitric oxygen, the implementation of plans for early enteral nutrition, and the incorporation of breastfeeding. These strategies have proven to be effective in other countries where mortality has decreased and where the quality of life of surviving infants has improved [1, 4]. 

As reported for other countries, we observed a higher neonatal mortality in the early stage of newborn life, particularly within the first 24 hours [1, 2, 11, 13, 17]. The stability of neonates that weigh less than 750 g is critical, and instability may cause approximately 80% of the deaths. Whether to resuscitate these neonates has been a controversial issue because of high hospital costs in addition to a high risk for later complications [4]. 

Currently, we are working to decrease these risks and to improve the quality of life as well as to evaluate late morbidity. With regard to the need for additional improvements, Figure 2 shows that the length of stay in the hospital ranged from 80 to 110 days for most of the neonates, with a minimal reduction during the study. The main goal, as documented in other studies, is to release the neonate in the best condition to his/her home to reduce the risk of rehospitalization [4, 17, 18]. 

In this study, an important objective (with medical and legal implications) was to determine the possible viability limits of ELBW neonates in hospitals that usually work with medical insurance services. We showed that the viability margins or limits considered during situations in which the survival was higher than 50% were reduced in the most recently studied 5-year period. Although the viability limit from this study does not present any legal implications at the present time, the limit (gestational age ≥25 weeks and birth weight ≥650 g) could be a clinical indicator to confer with the parents on making medical decisions. Since 2000, in our NICU, all newborns have a standardized neonatal intensive care protocol, regardless of their birth weight or gestational age; so, we think the mortality rate has not been influenced by discontinuation of intensive care because of poor prognosis. 

The strengths of this study included its review of a 25-year span of time, using an acceptable sample, and having more than 95% of the data available to study ELBW neonates. Of course, this study may not be representative of all private hospitals in Mexico, and there may have been selection bias because this hospital was chosen by mothers with better health and higher socioeconomic status; also, when consider weight groups, they were small. However, we have provided additional information to help the Beneficencia Española face the challenge of maintaining a first-level center for these patients; therefore, it is necessary to communicate the current implications of caring for ELBW neonates. Future work will need to address the survival conditions in the medium and long terms because mortality is not the only quality indicator of improvement at NICU's; for instance, in 35 patients followed up in the period 2000–2009, the main chronic conditions were bronchopulmonary dysplasia and intracranial hemorrhage (Table 4). 

## 11. Conclusions

Early- and medium-term mortality in neonates has significantly decreased in our hospital since 1995, particularly in the last 5-year period without a modification in the length of hospital stay. The viability limits have been reduced, and there is a high probability of survival for 650 g neonates or those equal or greater to 26 weeks of gestation.

## Figures and Tables

**Table 1:** Characteristics of ELBW neonates across 5-year periods.

		1985–1989	1990–1994	1995–1999	2000–2004	2005–2009	*P*-Value
		*n* = 43	*n* = 22	*n* = 16	*n* = 20	*n* = 57	
Sex							
	Male	23 (53.5%)	10 (45.5%)	8 (50%)	9 (45%)	25 (43.8%)	*.90*
	Female	20 (46.5%)	12 (54.5%)	8 (50%)	11 (55%)	32 (56.2%)	

*Gestation*							
	Single	21 (48.8%)	14 (63.6%)	10 (62.5%)	14 (70%)	34 (59.6%)	*.09*
	Twin	22 (51.2%)	5 (22.7%)	5 (31.3%)	6 (30%)	21 (36.8%)	
	Multiple	0	3 (13.6%)	1 (6.3%)	0	2 (3.6%)	

*Weeks of gestational age*							
21		1	0	0	1	2	*.17*
22		1	1	0	0	1	
23		4	1	2	2	3	
24		2	3	0	3	4	
	21–24	8 (18.6%)	5 (22.7%)	2(12.5%)	6 (30%)	10 (17.5%)	
25		5	3	2	1	7	
26		7	2	2	0	4	
27		6	1	0	3	5	
28		4	2	3	2	7	
29		10	2	1	4	5	
30		4	3	5	4	9	
	25–30	34 (79.1%)	13(59.1%)	13(81.3%)	14 (70%)	37 (64.9%)	
31		0	1	0	0	2	
32		1	1	0	0	3	
34		0	1	0	0	4	
	31–34	1 (2.3%)	13(13.6%)	0 (0%)	0 (0%)	9 (15.7%)	
35		0	1	0	0	1	
37		0	0	1	0	0	
	36–37	(0%)	1 (4.5%)	1 (6.3%)	0 (0%)	1 (2.5%)	

*Transfers*							
	<8 days	3 (6.9%)	0	0	2 (10%)	1 (2.5%)	.28
	≥8 days	0	0	0	0	1 (2.5%)	

*Chi-Square Pearson test.

**Table 2:** Early-neonatal, neonatal, and 120-day mortality in ELBW neonates by 5-year periods: 1985–2008.

Birth weight	1985–1989 *n* = 43# dead/# total (%)	1990–1994 *n* = 22# dead/# total (%)	1995–1999 *n* = 16# dead/# total (%)	2000–2004 *n* = 20# dead/# total (%)	2005–2009 *n* = 57# dead/# total (%)	*P*-value
*Early-neonatal mortality (*≤*8 days)*						
751–1000 g	11/23^§^ (47.0%)	6/14 (42.9%)	2/13 (15.4%)	2/7^§^ (28.5%)	2/28 (7.1%)	*.0008*
500–750 g	15/18 (83.3%)	7/8 (87.5%)	2/3 (66.7%)	5/10^§^ (50.0%)	9/23^§^ (39.1%)	*.0004*
<500 g	1/1 (100%)	—	—	1/1 (100%)	3/5 (60.0%)	—
*Total*	*27 (62.8)*%	*13 (59.1)*%	*4 (25)*%	*7 (35)*%	*14 (24.5)*%	< *.0001*

*Neonatal mortality (*≤*28 days)*						
751–1000 g	12/23^§^ (52.1%)	7/14 (50.0%)	5/13 (38.5%)	3/7^§^ (42.8%)	3/28 (10.7%)	*.001*
500–750 g	16/18 (88.9%)	7/8 (87.5%)	3/3 (100%)	6/10^§^ (60.0%)	13/23^§^ (56.5%)	*.005*
<500 g	1/1 (100%)	—	—	1/1 (100%)	3/5 (60.0%)	—
*Total*	*29 (67.4)*%	*14 (63.6)*%	*8 (50)*%	*9 (45)*%	*19 (33.3)*%	*.0003*

* 120-day mortality*						
751–1000 g	15/23^§^ (65.2%)	8/14 (57.1%)	6/13 (46.2%)	3/7^§^ (42.8%)	4/27^§^ (14.3%)	*.0002*
500–750 g	16/18 (88.9%)	7/8 (87.5%)	3/3 (100%)	6/10^§^ (60.0%)	14/23^§^ (60.8%)	*.01*
<500 g	1/1 (100%)	—	—	1/1 (100%)	4/5 (80.0%)	—
*Total*	*32 (74.4)*%	*15 (68.2)*%	*9 (56.3)*%	*9 (45)*%	*22 (38)*%	< *.0001*

*Chi-square linear-by-linear association tests. ^§^Includes transferred patients.

**Table 3:** Preterm mortality rates in different countries.

Country	*n*	Mortality rates*	Year(s)	Mortality rate stratified by birth weight (in grams)		
				<500	501–750	751–1000
Japan^1^	3065	Neonatal	2005	47.5%	16.1%	6.5%
		NICU	2005	57.5%	21.6%	8%
United Kingdom and USA^2^	4172	DR	1996–2000	52%	—	—
		NICU		62%	—	—
Israel^5^<751 g	97	NICU hospital inborn	2003–2006	44%		—
		NICU hospital outborn		36%		—
USA^4^	8312	120 days	1997–2002	—	45%	12%
Mexico (Private hospital)	40	Early neonatal	2005–2009	60%	39.1%	7.1%
		Neonatal		60%	56.5%	10.7%
		120-day		80%	60.8%	14.3%
Germany^15^<1000 g	8677	NICU	2000–2005		15%	
Finland^13^	529511	Neonatal	1996–1997	89%	55%	23%
			1999–2000	75%	45%	16%
Spain^16^	28	Early neonatal	2000–2003	—	11.1%	15.7%
		Neonatal		—	33.3%	21%
Turkey^17^	135	NICU	1997–2000	—	91.7%	21.1%
Uruguay^11^	130	NICU	2001–2004	100%	80.9%	25%
Mexico (Public System)^10^	250	Early neonatal	1999–2001	81.8%	78.2%	26.6%
Norway^18^	638	NICU	1999–2000	90%	58%	28%
India^7^	137	NICU	1994–2000	100%	77%	39%
Thailand^8^	22	Neonatal	2003–2006	—	80%	48%

*Early neonatal (≤8 days), neonatal (≤28 days) NICU (Neonatal Intensive Care Unit), DR (Delivery Room), 120-day (survival of ≤120 days). Inborn: newborn born in their hospital, outborn: newborn born in other hospital.

**Table 4:** Morbidity in 35 infants with birth weight <1000 grams: 2005–2009.

Diagnosis	*n*	(%)
Bronchopulmonary dysplasia	7	(20%)
Intracranial hemorrhage (III-IV)	7	(20%)
Retinopathy (III-IV)	3	(8.5%)
Hydrocephaly	2	(5.7%)
Short bowel syndrome	1	(2.8%)
